# Association between individual fat depots and cardio-metabolic traits in normal- and overweight children, adolescents and adults

**DOI:** 10.1038/nutd.2017.20

**Published:** 2017-05-08

**Authors:** M Hübers, C Geisler, S Plachta-Danielzik, M J Müller

**Affiliations:** 1 Institute of Human Nutrition and Food Science, Christian-Albrechts-University, Kiel, Germany; 2 Institute of Epidemiology, Christian-Albrechts-University, Kiel, Germany

## Abstract

**Objectives:**

To determine age-related associations between fat mass (FM), regional fat depots and cardiometabolic traits in normal- and overweight children, adolescents and adults.

**Methods:**

Detailed body composition (regional subcutaneous and visceral adipose tissue; SAT, VAT) by whole-body magnetic resonance imaging (MRI), FM and fat-free mass by air-displacement plethysmography, systolic and diastolic blood pressure (SBP, DBP), triglycerides (TG), high-density lipoprotein cholesterol (HDL), plasma glucose and plasma insulin were measured in 433 subjects (BMI: 23.6 (21.0–27.7); 151 children and adolescents, aged 6–18 years, 150 young adults, aged 18–30 years and 132 adults, aged 30–60 years). Data were derived from pooled data of the ‘Reference Center for Body Composition’ in Kiel, Germany. Insulin resistance was determined by the homeostatic model assessment of insulin resistance (HOMA-IR). Partial correlations and multivariate linear regression analyses were used to evaluate the associations between body composition and cardiometabolic traits. A descriptive approach was used to demonstrate age-dependent differences in associations between body fat depots and insulin resistance, independent of BMI.

**Results:**

FM, SAT, and VAT increased from childhood to adulthood with low VAT in children and adolescents. When compared to children, TG was higher in adults. HDL and DBP did not differ between age groups. Insulin resistance was highest in male adolescents and female young adults. Associations between body fat depots and cardiometabolic traits were seen after puberty with no associations in pre- and intrapubertal children. When compared to FM, SAT and VAT had the strongest association with insulin resistance in adults. This association was independent of BMI.

**Conclusions:**

Associations between individual body fat depots and most cardiometabolic traits became evident after puberty only. The strongest associations were observed between insulin resistance and abdominal fat in adults. The impact of VAT was independent of BMI.

## Introduction

To address the impact of overweight on cardiometabolic risk most studies are based on body mass index (BMI).^1, 2, 3^ However, BMI can neither differentiate between fat mass (FM) and fat-free mass (FFM) nor give detailed information about body fat distribution.^4, 5^ To go beyond BMI, detailed body composition and appropriate modeling taking into account body function have to be considered.^6^ Magnetic resonance imaging (MRI) and computed tomography are the gold-standard methods to determine trunk and peripheral subcutaneous adipose tissue (SAT) and visceral adipose tissue (VAT). In children and adolescents MRI is the most suitable measurement because it does not expose them to radiation.

Our present knowledge about the association between individual body fat depots and cardio-metabolic risk factors is mainly based on studies on adults. In addition, there is only limited knowledge about regional fat depots in children and adolescents. In a cross-sectional study SAT and VAT were lower in children compared to adults.^7^ In a longitudinal study, SAT increased during adolescence, whereas VAT remained low.^8^ By contrast, both, SAT and VAT, increased between age of 11 and 13 years.^9^ With regard to gender differences, girls compared with boys had greater masses of VAT and SAT.^10^ This was contrary to other authors who observed that VAT was greater in boys than in girls.^11^

In obese adolescents and adults cardiometabolic traits and insulin resistance have been mainly related to VAT and liver fat.^12, 13, 14, 15, 16^ In children and adolescents the degree of insulin resistance rises from pre- to intrapuberty and decreases after puberty.^17, 18^ However, the association between cardiometabolic traits and insulin resistance with detailed body composition has not been well characterized in children and adolescents. As in adults, SAT and VAT were differently associated with cardiometabolic traits in children and adolescents.^19, 20, 21, 22^ Both, SAT and VAT were independently correlated with blood pressure,^19^ whereas VAT was positively associated with plasma triglycerides (TG) and negatively associated with high-density lipoprotein cholesterol (HDL).^20^ In addition, there was a positive relationship between either VAT or SAT and insulin resistance.^20, 21, 22, 23^

Untill now, there is no systematic study of the associations between individual body fat depots and cardiometabolic risk in different age groups. The aim of this study was to determine the associations between MRI-derived individual fat depots and cardiometabolic traits in children and adolescents compared to young and older adults. Furthermore, we investigated the associations between insulin resistance, relative to BMI, and regional fat depots.

## Methods

All studies had been approved by the Ethical Committee of Christian-Albrechts-University of Kiel, Germany, and conducted according to the guidelines laid down in the ‘Declaration of Helsinki’. Written informed consent was obtained from each subject and, in state of minority, from its legal guardian before participation.

### Subjects

This investigation was a secondary analysis of data collected at the ‘Reference Center for Body Composition’ (Institute of Human Nutrition and Food Science at the University of Kiel, Germany) between 2005 and 2016. In this cross sectional study, data of 433 healthy Caucasian subjects (151 children and adolescents, aged 6–18 years, 150 young adults, aged 18–30 years and 132 adults, aged 30–60 years) were analyzed. Exclusion criteria were metallic implants, smoking, pregnancy, chronic or acute diseases, and medication intake that influences body composition. Subjects were recruited by notice boards postings and advertisements in the local newspaper. Study measurements of anthropometry, body composition, detailed body composition by whole body MRI and cardiometabolic variables were performed in children, adolescents and adults. Self-assessment of pubertal stage was determined according to the definition of Marshall and Tanner.^24^ On the basis of breast and genital stages pubertal status was categorized into three groups (prepubertal: Tanner I; intrapubertal: Tanner II–III, postpubertal: Tanner⩾IV).^18^ In this analysis prepubertal and intrapubertal subjects were grouped together. This was done because they did not differ in associations between body composition and cardiometabolic traits (data not shown), and because of a low number of prepubertal children (*n*=26).

### Anthropometric measures

Body weight was measured to the nearest 0.01 kg using an electronic Tanita scale coupled to the BOD POD Body Composition Tracking System (Life Measurement Instruments, Concord, CA, USA). Body height was assessed to the nearest 0.5 cm by using a stadiometer (SECA, Modell 220, Hamburg, Germany). BMI was calculated as body weight (kg)/body height (m)^2^. To define overweight and obesity for children and adolescents (>90th percentile; >97th percentile) German age- and sex-specific references were used.^25^ Waist circumference (WC) was measured midway between the lowest rib and the top of the iliac crest with the subjects standing in upright position. The measurement was made with a nonelastic plastic tape placed parallel to the floor at the end of a normal expiration.

### Densitometry

Air-displacement plethysmography was perfomed by BOD POD device to assess body composition. Child-specific equations were used to determine FM, as in detail described elsewhere.^26^ In adults, FM was calculated using the equation by Siri *et al.*^27^ FFM was then calculated from the difference between body weight and FM. Because of reported difficulties in BOD POD calibration in one of the children studies cross-validated gender specific equations of FM were developed for children and adolescents, as procedure is described elsewhere.^28^ Around 222 children and adolescents were randomly split into two parts; 155 (91 female, 64 male) were assigned to the derivation group, with 67 (31 female, 36 male) subjects in the cross-validation group. When compared to derivation group, the cross-validation group did not differ in age, gender, pubertal status, height, weight, BMI, WC, and FM measured by bio-impedance analysis. The correlation coefficients between FM measured by bio-impedance analysis and predicted FM based on BOD POD algorithm did not differ between the two groups. Following equations based on stepwise multiple regression analysis were developed for children and adolescents:


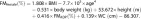



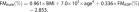


On the basis of calculated FM and FFM, FM-Index (FMI) and FFM-Index (FFMI) were calculated for children and adolescents as FM (kg)/height (m)^*n*^ and FFM (kg)/height (m)^*n*^. Height-independent FM is confounded by age in adolescents, whereas age was not included as a significant independent variable in children. By contrast, height-independent FFM is confounded by age in children but not in adolescents. Age-adjusted power of height (*n*) was determined using log–log regression analysis^29^ (FMI: *n*=6.1, 2.3 and FFMI: *n*=2.3, 2.9, respectively for children and adolescents). In adults FMI and FFMI were calculated, as FM (kg)/height (m)^2^ and FFM (kg)/height (m)^2^.

### Magnetic resonance imaging (MRI)

As described previously, detailed body composition of all subjects was performed by using whole-body MRI with a 1.5T scanner (Magnetom Vision or Magnetom Avanto, Siemens Medical Systems, Erlangen, Germany).^30, 31^ Briefly, participants were examined in a supine position with their arms extended above their heads and they were asked to hold their breath during measurement of abdominal and thoracic regions for image acquisition. In the present analysis volumes of SAT and VAT were manually segmented by using segmentation software (SliceOmatic 4.3 and 5.0, Tomovision, Montreal, Canada). SAT was analyzed from ankle to wrist. In this case SAT_legs_ were defined from ankle to femoral heads and SAT_arms_ were defined from humerus heads to wrist. Between femoral and humerus heads volume of SAT_trunk_ was assessed. VAT was defined as intra-abdominal fat between femoral heads and the top of the liver. Volumes of SAT and VAT were determined by the sum of all adipose tissue areas (cm^2^) multiplied by slice thickness. Volume data were then transformed into tissue masses by using the following density: 0.923 g cm^−3^ for SAT and VAT.

### Cardiometabolic risk factors

Systolic and diastolic blood pressure were measured by manual sphygmomanometer in a seated position. After an 8-h overnight fast blood samples were taken and analyzed by standard procedures. HDL was assessed enzymatically by Konelab-20i-Analyzer (Konelab, Espoo, Finland). Triglyceride and plasma glucose were analyzed enzymatically using a Konelab-Test-Kit (Thermo Clinical Labsystems, Frankfurt, Germany). Plasma insulin was measured by radioimmunoassay (Adaltis, Freiburg, Germany). Insulin resistance was then calculated by homeostasis model assessment of insulin resistance (HOMA-IR):

HOMA-IR=plasma glucose (mmol/L)xplasma insulin (μU/ml)/22.5.^32^

### Statistical analysis

Statistical analyses were performed with SPSS statistical software (SPSS 24.0, Inc., Chicago, Illinois, USA). All data are given as median and interquartil range because of not normally distributed data. Kruskal-wallis test with bonferroni correction was performed for more than two groups (pre- and intrapubertal subjects, postpubertal subjects, young adults and adults). To examine the association of individual body components on cardiometabolic traits, age- and gender-adjusted partial correlations were performed. Differences in correlation coefficients between age groups were tested by using the method of Eid, Gollwitzer, Schmitt.^33^ Further, stepwise multivariate linear regression analyses were done. Four models were conducted. Model 1 included FMI, Model 2 SAT_trunk_, Model 3 VAT and Model 4 SAT_trunk_ and VAT as independent variables. All models were adjusted for age and gender. In addition, a descriptive approach was used to demonstrate age-dependent differences in individual body components between three different HOMA-IR per BMI groups (low, normal and high). These three groups were stratified according to their age dependent deviation from the regression line. Cut-offs were chosen from tertiles between HOMA-IR and BMI (low: residuals<−0.48,<−0.90,<−0.92,<−0.69; normal: residuals⩽0.16 and⩾−0.48,⩽0.25 and⩾−0.90,⩽0.00 and⩾−0.92,⩽0.29 and⩾−0.69; high: residuals>0.16,>0.25,>0.00, >0.29, respectively for children, adolescents, young adults, and adults). All tests were two-sided and level of significance was set at *P*<0.05.

## Results

### Detailed body composition: differences between age groups

The characteristics of the study population are shown in Table 1. When compared with children, height, weight, FM, and FFM were significantly higher in adolescents and with no significant differences to adults. When compared with young adults, VAT was lower in children and adolescents. In males, VAT was significantly higher in older compared with young adults. In females, SAT_total_, SAT_arms_, and SAT_trunk_ were higher in young adults compared with children and adolescents. By contrast, SAT_trunk_ was the only subcutaneous fat depot in males which was significantly higher in young adults compared to children and adolescents.

### Cardiometabolic risks: Differences between age groups

In females, plasma insulin and HOMA-IR were highest in young adults when compared to other age groups (Table 1). In males, plasma insulin and HOMA-IR were highest in adolescents. In both genders, HOMA-IR did not differ between adolescents and adults. There were no differences in SBP, DBP and HDL between the different age groups, except for SBP in male adolescents, and high TG level in female young adults and male adults.

### Associations between body composition and cardiometabolic traits: Differences between age groups

Age- and gender-adjusted partial correlation coefficients between individual body fat depots and cardiometabolic traits are shown in Table 2. In general, correlation coefficients were low to moderate. As for SBP, DBP, TG and plasma glucose, significant associations were seen after puberty only. The associations between SAT_trunk_ and either SBP or DBP or TG did not differ in adolescents, young adults and adults. Strongest associations were observed between plasma insulin levels or HOMA-IR and truncal fat depots (SAT_trunk_, VAT). When compared with adults, the associations between individual body fat depots and HDL were higher in children and adolescents.

Using stepwise multivariate linear regression analysis, the explained variance and respective beta coefficients of cardiometabolic traits as dependent variables and FMI, SAT_trunk_, VAT and the combination of SAT_trunk_ and VAT, as independent variables are shown in Table 3. The explained variance of cardiometabolic traits was moderate, maximally, 35.2% of the variance of HOMA-IR was explained by SAT_trunk_ and VAT in adults.

When compared with adolescents and adults, the explained variances of SBP, DBP, TG and plasma glucose by body fat depots were low (⩽5.3%) in children. However, the explained variances of HOMA-IR and plasma insulin by parameters of body composition differed only slightly between children, adolescents and adults. The explained variance of HDL by body fat depots decreased from children to adults.

When compared to FMI, the explained variance of plasma insulin and HOMA-IR by SAT_trunk_ and VAT were moderately increased in young and older adults. When compared to FMI, SAT_trunk_ and VAT did not exceed the explained variance of HDL, whereas abdominal fat depots added to the variance of TG in adolescents.

### Association between detailed body composition and insulin resistance

Investigating subjects with low HOMA-IR (insulin sensitive), normal HOMA-IR or high HOMA-IR (insulin resistance) relative to their BMI, age-dependent differences in individual body components between these groups became evident (Table 4). In children and adolescents, no differences were found in body fat depots between groups differing in insulin resistance. By contrast, in young adults, adipose tissue, SAT_total_, SAT_trunk_ and SAT_legs_ were significantly higher in insulin resistant subjects. In older adults, VAT was highest in insulin resistant subjects.

## Discussion

Up to now, there are limited data on the association between regional fat depots and metabolic disturbance in normal- and overweight children and adolescents compared with adults.^34, 35^ To our knowledge, the present study is first to systematically investigate detailed body composition by age group and gender as well as its associations to cardiometabolic traits in 6–60 year old subjects.

### Detailed body Composition in different age groups

In both genders, VAT was low in children and adolescents compared with adults (Table 1). This is in line with previous data.^7, 8^ However, other authors reported that during childhood, growth of VAT decelerated with increasing age.^34^ In our study sexual dimorphism was apparent after puberty only with males having more VAT than females. By contrast, other authors observed higher masses of VAT in girls compared to boys.^10^

### Cardiometabolic risks in different age groups

As shown in Table 1, in males, insulin resistance increased from pre- and intrapuberty to postpuberty, with changes to young adulthood in females only. These findings support previous data.^36, 37^ By contrast, other authors reported that the increase in insulin resistance from childhood to adulthood was independent of gender.^38^ Studies on blood pressure showed that SBP and DBP increased with age.^38, 39^ These results are contrary to our findings.

### Associations between body composition and cardiometabolic traits in different age groups

Low and moderate associations were observed between cardiometabolic traits and body fat depots in normal- and overweight children, adolescents and adults (Table 2). This is in line with previous data.^38^ However, moderate associations were seen after puberty only. These findings indicate that in pre- and intrapubertal children individual body components are not related to cardiometabolic risk, as it is in older subjects. Accordingly, a study on pre- and intrapubertal children found no associations between abdominal fat depots vs blood pressure and blood lipids in European Americans.^40^ Positive associations became evident between SAT_trunk_, VAT, and cardiometabolic traits in adolescents.^19^

Overall, there was a limited explained variance of cardiometabolic traits by FMI and different regional fat depots (Table 3). When compared to FMI, abdominal fat depots, especially VAT, were stronger associated to insulin resistance in adults than in children and adolescents. The associations between HDL and body fat depots decreased with age. As HDL is positively associated to physical activity in children, adolescents and adults,^41, 42^ a possible explanation may be increasing sedentary behavior with age which is due to school entry and entering the workplace.^43, 44^ This is in line with the finding that children improved HDL by increased physical activity which was independent of weight change.^45^

### Associations between detailed body composition and insulin resistance

To address the role of individual body fat depots on insulin resistance, independent of BMI, differences in age groups became evident (Table 4). In children and adolescents, there were no differences in body fat depots between groups differing in insulin resistance. By contrast, regional SAT depots were significantly different in young adults, with VAT as associated to insulin resistance in older adults only. Our findings suggest that VAT becomes an important determinant of insulin resistance as age increased to adulthood. In our study, VAT was very low in children and adolescents and started to increase in young adults (Table 1). This is in line with other data that VAT is a primary determinant of insulin resistance in adults only.^46, 47, 48^

### Study strengths and limitations

To our knowledge, this is the first study to systematically investigate age-related associations between individual body components and cardiometabolic traits in children and adolescents compared to adults. Detailed body composition was measured by MRI. This is the gold-standard method of body composition. We have used two different approaches. First, an analytic approach was chosen to examine age-dependent associations between cardiometabolic traits and body fat depots. Second, a descriptive approach was conducted to investigate age-dependent differences in body fat depots in groups differing in insulin resistance. A limitation of this study is that we have used cross-sectional data only. Thus, no causal relationships can be derived from our data.

## Conclusion

Associations between regional fat depots and cardiometabolic traits were seen after puberty only. The impact of FMI on cardiometabolic traits is limited. With regard to HOMA-IR, this was improved by measuring individual fat depots in adults. The strongest associations were observed between abdominal fat depots and insulin resistance. When compared with adults, VAT was low in normal- and overweight children and adolescents. VAT has no association with insulin resistance groups in children, adolescents and young adults. The role of VAT is apparent in adults only.

## Figures and Tables

**Table 1:** Characteristics of the study population stratified by age group and gender

*Age groups*^a^	*(1) Children*		*(2) Adolescents*		*(3) Young Adults*		*(4) Adults*	
	*Females*	*Males*	*Females*	*Males*	*Females*	*Males*	*Females*	*Males*
*n*	37	43	41	30	69	81	69	63
Age (y)	10.9 (9.4–13.4)	12.3 (9.4–14.1)	15.8 (15.3–16.3)	16.3 (15.6–17.3)	26.0 (24.0–26.0)^b,c^	25.0 (23.0–27.0)^b,c^	44.0 (36.0–50.0)^b,c,d^	41.0 (35.0–50.0)^b,c,d^
Height (m)	1.49 (1.34–1.61)	1.58 (1.42–1.70)	1.68 (1.65–1.72)^b^	1.78 (1.73–1.83)^b^	1.70 (1.65–1.75)^b^	1.81 (1.76–1.87)^b^	1.67 (1.63–1.72)^b^	1.79 (1.76–1.84)^b^
Weight (kg)	43.4 (29.4–56.9)	53.1 (32.3–72.5)	65.6 (55.0–80.7)^b^	73.0 (64.0–101.6)^b^	69.5 (61.0–87.2)^b^	78.6 (71.1–85.1)^b^	68.0 (61.4–79.6)^b^	81.2 (73.5–92.3)^b^
OW (OB) (%)	35.1 (27.0)	34.9 (16.3)	43.9 (36.6)	43.3 (36.7)	37.7 (29.0)	33.3 (8.6)	40.6 (18.8)	46.0 (17.5)
BMI (kg/m^2^)	19.3 (16.5–25.5)	19.0 (15.7–24.8)	21.8 (19.8–29.6)	23.4 (20.0–31.1)^b^	23.9 (21.7–31.7)^b^	23.4 (22.1–25.7)^b^	24.3 (21.7–27.7)^b^	24.5 (23.2–28.7)^b^
FM (kg)	9.4 (4.7–19.9)	9.9 (5.0–19.4)	16.8 (12.4–31.9)^b^	14.0 (9.3–29.6)^b^	22.1 (17.4–37.8)^b^	13.2 (10.1–18.9)	23.4 (16.7–30.5)^b^	17.2 (12.9–24.4)^b^
FFM (kg)	32.5 (24.5–39.3)	40.6 (27.4–50.0)	47.6 (41.6–51.1)^b^	57.7 (54.8–68.2)^b^	47.5 (43.6–52.0)^b^	64.5 (59.0–68.7)^b^	45.0 (41.8–50.2)^b^	65.3 (59.4–69.3)^b^
FMI (kg/m^e^)	0.95 (0.55–1.56)	0.70 (0.48–0.98)	4.8 (3.9–10.2)	4.0 (2.6–8.2)	7.8 (6.0–14.2)	3.8 (3.0–5.8)	8.3 (6.3–10.9)	5.3 (3.9–7.3)
FFMI (kg/m^e^)	13.0 (12.1–14.5)	12.9 (11.8–15.1)	10.1 (9.3–11.4)	11.1 (10.5–12.0)	16.4 (15.5–17.9)	19.6 (18.5–20.5)	16.0 (15.0–17.8)	19.9 (18.7–21.5)
Adipose tissue (kg)	9.5 (7.1–19.3)	9.0 (4.7–18.5)	15.0 (11.9–29.1)	11.6 (8.4–25.0)	21.5 (17.5–34.6)^b^	12.9 (10.8–18.6)^b^	22.6 (17.0–30.8)^b^	18.2 (14.0–24.5)^b,d^
SAT total (kg)	9.1 (6.9–18.8)	8.7 (4.6–17.9)	14.5 (11.5–27.9)	11.3 (8.1–23.2)	20.0 (17.0–33.3)^b^	11.8 (10.2–17.2)	21.4 (16.1–28.3)^b^	14.8 (11.7–18.6)^b^
SAT arms (kg)	1.2 (1.0–2.2)	1.2 (0.7–2.1)	1.7 (1.4–3.2)	1.4 (1.1–3.0)	2.2 (1.8–3.6)^b^	1.5 (1.4–2.2)	2.3 (1.7–3.0)^b^	1.9 (1.6–2.2)^b^
SAT trunk (kg)	2.9 (2.1–6.3)	2.6 (1.3–6.7)	4.7 (3.7–13.5)	3.8 (2.8–10.4)	8.6 (6.2–14.3)^b^	4.7 (3.7–8.1)^b^	9.1 (6.3–13.3)^b^	6.8 (4.9–9.0)^b^
SAT legs (kg)	5.3 (3.8–8.7)	4.6 (2.6–8.4)	8.0 (6.4–12.5)^b^	6.0 (4.4–11.0)	9.8 (8.2–13.9)^b^	5.6 (4.8–7.3)	8.9 (7.0–11.9)^b^	6.3 (5.3–7.7)^b^
VAT (kg)	0.36 (0.22–0.87)	0.29 (0.16–0.88)	0.47 (0.34–0.96)	0.64 (0.37–1.30)	1.11 (0.60–1.71)^b,c^	1.13 (0.70–2.15)^b^	1.36 (0.68–2.29)^b,c^	2.50 (1.69–5.17)^b,c,d^
SBP (mm Hg)	120 (108–121)	120 (110–125)	118 (110–120)	128 (120–134)^b^	115 (110–125)	120 (110–125)^c^	118 (110–125)	120 (115–130)
DBP (mm Hg)	70 (70–80)	75 (70–80)	75 (70–80)	76 (70–85)	80 (70–82)	80 (75–85)	80 (70–80)	80 (75–85)
TG (mg/dl)	75 (59–96)	67 (47–121)	85 (70–104)	86 (69–114)	102 (73–145)^b^	91 (70–127)^b^	86 (70–108)	106 (78–143)^b^
HDL (mg/dl)	54 (46–60)	53 (46–62)	52 (43–66)	51 (42–59)	55 (46–72)	51 (40–57)	65 (48–73)	46 (33–58)
Glucose (mg/dl)	89 (84–94)	90 (85–96)	87 (82–93)	91 (86–94)	88 (83–92)	86 (78–94)	94 (86–99)^c,d^	99 (94–105)^b,c,d^
Insulin (μU/ml)	8.7 (4.9–11.1)	7.7 (4.4–11.6)	12.1 (6.9–15.3)	10.7 (6.9–14.5)	12.5 (8.0–18.6)^b^	7.8 (6.3–10.1)	9.2 (6.8–12.3)^d^	7.7 (5.9–14.1)
HOMA-IR	1.92 (0.98–2.36)	1.84 (0.92–2.79)	2.49 (1.46–3.11)	2.34 (1.46–3.08)	2.68 (1.62–4.34)^b^	1.64 (1.20–2.15)	2.20 (1.53–2.84)	1.98 (1.40–3.31)

Abbreviations: DBP, diastolic blood pressure; FFM, fat-free mass; FFMI, fat-free mass index; FM, fat mass; FMI, fat mass index; HDL, high-density lipoprotein cholesterol; OB, obese; OW, overweight; SAT, subcutaneous adipose tissue; SBP, systolic blood pressure; TG, triglyceride; VAT, visceral adipose tissue.

a (1), pre- and intrapubertal children; (2), postpubertal adolescents; (3), young adults (18–30 years old); (4), adults (30–60 years old).

b Significantly different from gender-dependent pre- and intrapubertal subjects.

c Significantly different from gender-dependent postpubertal subjects.

d Significantly different from gender-dependent young adults (Kruskal-wallis test with bonferroni correction, *P*<0.05).

e Power of height which is unequal in pre-and intrapubertal, postpubertal and adult stage (FMI: 6.1, 2.3, 2.0, 2.0; FFMI: 2.3, 2.9, 2.0, 2.0, respectively).

Data are given as median and interquartil range.

**Table 2:** Age- and gender-adjusted partial correlations between parameters of body composition and cardiometabolic traits in children, adolescents and adults

*Age groups*^a^	*Correlation coefficient (r)*																			
	*FMI*				*SAT*_*arms*_				*SAT*_*trunk*_				*SAT*_*legs*_				*VAT*			
	*1*	*2*	*3*	*4*	*1*	*2*	*3*	*4*	*1*	*2*	*3*	*4*	*1*	*2*	*3*	*4*	*1*	*2*	*3*	*4*
*n*	80	71	150	132	80	71	150	132	80	71	150	132	80	71	150	132	80	71	150	132
SBP	0.17^b,c^	**0.31**	**0.43**	**0.42**	**0.29**	**0.27**	**0.39**	**0.27**	**0.25**	**0.31**	**0.43**	**0.42**	**0.29**	**0.28**	**0.36**	**0.30**	0.19^c^	**0.27**^c^	**0.27**^c^	**0.48**
DBP	0.14^b,c,d^	**0.42**	**0.41**	**0.37**	**0.33**	**0.43**	**0.35**	**0.25**	**0.29**	**0.42**	**0.38**	**0.40**	**0.30**	**0.37**	**0.34**	**0.23**	**0.24**^c^	**0.42**	**0.26**^c^	**0.45**
TG	0.00^b,c,d^	**0.37**	**0.35**	**0.24**	−0.05^b,d^	**0.34**	**0.31**	0.16	0.02^b,d^	**0.34**	**0.37**	**0.24**	−0.12^b,c,d^	**0.29**	**0.26**	0.12	0.16^c,d^	**0.54**	**0.36**	**0.41**
HDL	−**0.53**^b,c^	−**0.42**	−**0.27**	−**0.31**	−**0.50**^b,c^	−**0.39**	−**0.26**	−**0.24**	−**0.52**^b^	−**0.41**	−**0.28**	−**0.33**	−**0.52**^b,c^	−**0.33**	−**0.21**	−**0.22**	−**0.50**^b,c^	−**0.37**	−**0.29**	−**0.30**
Glucose	−0.15^c^	−**0.23**^b,c^	0.07^c^	**0.30**	−0.17^b,c^	−**0.25**^b,c^	0.15	**0.30**	−**0.24**^b,c^	−**0.27**^b,c^	**0.18**	**0.30**	−0.19^b,c^	−0.22^b,c^	0.05^c^	**0.33**	−**0.26**^b,c^	−0.17^b,c^	**0.35**	**0.37**
Insulin	**0.55**	**0.54**	**0.48**	**0.48**	**0.55**	**0.52**	**0.52**	**0.39**	**0.57**	**0.55**	**0.56**	**0.53**	**0.53**	**0.55**^c^	**0.37**	**0.34**	**0.64**	**0.55**	**0.53**	**0.50**
HOMA-IR	**0.51**	**0.51**	**0.45**	**0.51**	**0.52**	**0.48**	**0.50**	**0.41**	**0.52**	**0.51**	**0.54**	**0.55**	**0.50**	**0.52**	**0.34**	**0.37**	**0.59**	**0.52**	**0.53**	**0.55**

Abbreviations: DBP, diastolic blood pressure; FMI, fat mass index; HDL, high-density lipoprotein cholesterol; SAT, subcutaneous adipose tissue; SBP, systolic blood pressure; TG, triglyceride; VAT, visceral adipose tissue.

a 1, pre- and intrapubertal children; 2, postpubertal adolescents; 3, young adults (18–30 years old); 4, adults (30–60 years old).

b Significantly different from correlation coefficient in young adults.

c Significantly different from correlation coefficient in adults, *P*<0.05.

d Significantly different from correlation coefficient in postpubertal adolescents (method of Eid, Gollwitzer, Schmitt^33^).

Significant correlation coefficients in bold, *P*<0.05.

**Table 3:** Multivariate linear regression on cardiometabolic traits as dependent variables

*Independent variables*	***FMI***^a^								***SAT***_***trunk***_^a^								***VAT***^a^								***SAT***_***trunk***_*+**VAT***^a^							
*age groups*^b^	*1*		*2*		*3*		*4*		*1*		*2*		*3*		*4*		*1*		*2*		*3*		*4*		*1*		*2*		*3*		*4*	
	*R^2^*	*β*	*R^2^*	*β*	*R^2^*	*β*	*R^2^*	*β*	*R^2^*	*β*	*R^2^*	*β*	*R^2^*	*β*	*R^2^*	*β*	*R^2^*	*β*	*R^2^*	*β*	*R^2^*	*β*	*R^2^*	*β*	*R^2^*	*β*^c^	*R^2^*	*β*^c^	*R^2^*	*β*^c^	*R^2^*	*β*^c^
*n*	80		71		150		132		80		71		150		132		80		71		150		132		80		71		150		132	
SBP	**0.0**	0.18	**5.9**	0.27	**17.9**	0.50	**15.0**	0.44	**3.5**	0.26	**6.3**	0.27	**17.7**	0.47	**15.5**	0.41	**0.0**	0.20	**4.6**	0.24	**6.5**	0.28	**20.6**	0.53	**3.5**	0.02	**6.3**	0.02	**17.7**	−0.06	**19.4**	0.52
DBP	**0.0**	0.16	**15.7**	0.42	**16.1**	0.47	**12.4**	0.40	**5.3**	0.31	**16.7**	0.42	**13.6**	0.41	**14.8**	0.41	**4.2**	0.26	**16.1**	0.41	**6.3**	0.27	**19.0**	0.51	**5.3**	0.09	**16.7**	0.20	**13.6**	0.00	**18.4**	0.50
TG	**0.0**	0.00	**11.7**	0.36	**11.3**	0.40	**4.5**	0.25	**0.0**	0.02	**9.9**	0.33	**12.6**	0.40	**4.6**	0.24	**0.0**	0.17	**27.2**	0.53	**11.8**	0.37	**15.2**	0.46	**0.0**	0.17	**32.6**	0.96	**12.6**	0.20	**15.8**	0.47
HDL	**25.4**	−0.54	**15.1**	−0.41	**5.6**	−0.29	**6.8**	−0.30	**23.2**	−0.60	**14.7**	−0.40	**6.3**	−0.29	**7.6**	−0.30	**21.6**	−0.54	**12.2**	−0.37	**6.6**	−0.28	**6.7**	−0.31	**23.2**	−0.25	**14.7**	−0.09	**6.6**	−0.28	**7.6**	−0.17
Glucose	**0.0**	−0.14	**0.0**	−0.23	**0.0**	0.08	**7.1**	0.31	**3.7**	−0.27	**5.8**	−0.27	**2.7**	0.20	**7.1**	0.29	**4.8**	−0.28	**0.0**	−0.17	**11.5**	0.36	**11.0**	0.39	**4.8**	-0.28	**5.8**	0.24	**11.5**	0.36	**11.3**	0.40
Insulin	**19.4**	0.47	**27.4**	0.54	**18.5**	0.51	**22.8**	0.54	**20.7**	0.56	**29.1**	0.55	**25.6**	0.57	**27.9**	0.55	**26.4**	0.59	**29.3**	0.55	**22.3**	0.49	**24.8**	0.58	**26.4**	0.59	**29.3**	0.55	**28.2**	0.24	**31.0**	0.31
HOMA-IR	**16.3**	0.43	**24.4**	0.50	**16.3**	0.48	**25.6**	0.57	**16.8**	0.51	**24.9**	0.50	**24.4**	0.55	**30.0**	0.57	**21.2**	0.53	**26.7**	0.52	**23.3**	0.50	**29.8**	0.63	**21.2**	0.53	**26.7**	0.52	**27.9**	0.28	**35.2**	0.64

Abbreviations: DBP, diastolic blood pressure; FMI, fat mass index; HDL, high-density lipoprotein cholesterol; SAT, subcutaneous adipose tissue; SBP, systolic blood pressure; TG, triglyceride; VAT, visceral adipose tissue.

a Model with independent variable/variables in bold adjusted for age and gender *R*^2^, additional explained variance (%) by independent variable/variables which is/are in bold compared to the base model which includes age and gender.

b 1, pre- and intrapubertal children; 2, postpubertal adolescents; 3, young adults (18–30 years old); 4, adults (30–60 years old).

c β-coefficient of VAT.

**Table 4:** Differences in body fat depots between three groups (low HOMA-IR per BMI, normal HOMA-IR per BMI and high HOMA-IR per BMI) stratified by age group

	*HOMA-IR/BMI ↓*	*HOMA-IR/BMI =*	*HOMA-IR/BMI ↑*	*P-value*
*Children*				
*n*	26	27	26	
FM (kg)	14.8 (4.7–23.4)	7.1 (4.2–12.8)	10.7 (7.6–20.5)	**0.038**
FFM (kg)	36.7 (27.4–49.8)	28.4 (23.6–38.9)	42.1 (32.4–47.1)	**0.011**
Adipose tissue (kg)	11.9 (4.7–22.9)	8.3 (4.7–12.6)	9.8 (8.5–22.5)	0.111
SAT total (kg)	11.5 (4.5–22.1)	7.9 (4.5–12.0)	9.4 (8.3–21.9)	0.121
SAT arms (kg)	1.5 (0.7–2.5)	1.1 (0.7–1.7)	1.3 (1.0–2.3)	0.193
SAT trunk (kg)	3.9 (1.3–9.3)	2.6 (1.3–3.9)	3.1 (2.4–8.0)	0.132
SAT legs (kg)	6.1 (2.6–10.5)	4.5 (2.5–6.4)	5.1 (4.5–9.4)	0.102
VAT (kg)	0.53 (0.18–0.97)	0.29 (0.16–0.47)	0.36 (0.26–1.02)	0.171
HOMA-IR	0.84 (0.57–2.05)	1.60 (0.97–1.91)	2.74 (2.07–4.10)	**<0.001**
				
*Adolescents*				
n	23	24	23	
FM (kg)	24.2 (12.5–35.5)	15.5 (9.9–29.6)	13.5 (10.8–31.9)	0.174
FFM (kg)	56.9 (49.3–64.9)	50.4 (44.4–57.3)	49.3 (43.9–52.1)	0.058
Adipose tissue (kg)	22.4 (11.9–31.3)	12.0 (9.7–24.6)	13.3 (11.1–30.2)	0.222
SAT total (kg)	21.1 (11.6–30.1)	11.6 (9.3–23.2)	12.9 (10.6–24.4)	0.222
SAT arms (kg)	2.6 (1.3–3.8)	1.4 (1.2–2.8)	1.5 (1.3–3.1)	0.338
SAT trunk (kg)	8.1 (3.6–13.5)	3.8 (3.2–9.7)	4.5 (3.1–13.9)	0.190
SAT legs (kg)	10.0 (6.2–13.7)	6.9 (4.7–11.3)	6.8 (5.8–12.5)	0.265
VAT (kg)	0.72 (0.44–1.20)	0.43 (0.31–0.81)	0.51 (0.32–1.20)	0.306
HOMA-IR	1.62 (0.97–2.49)	2.02 (1.38–2.89)	3.25 (2.72–5.49)	**<0.001**
				
*Young adults*				
*n*	47	47	47	
FM (kg)	17.6 (12.3–37.8)	15.6 (10.3–19.7)	20.8 (14.8–26.1)	**0.034**
FFM (kg)	62.1 (49.4–68.7)	58.7 (50.3–65.4)	53.6 (44.3–63.1)	**0.010**
Adipose tissue (kg)	16.5 (11.1–34.6)	16.0 (11.0–19.6)	20.4 (14.8–26.1)	**0.013**
SAT total (kg)	15.2 (10.4–33.3)	14.4 (10.3–19.3)	18.9 (14.2–25.0)	**0.008**
SAT arms (kg)	1.9 (1.4–3.1)	1.7 (1.4–2.2)	2.2 (1.6–2.8)	0.066
SAT trunk (kg)	5.9 (3.8–14.6)	5.8 (3.8–8.1)	8.8 (6.2–12.0)	**0.006**
SAT legs (kg)	8.1 (5.2–13.9)	6.4 (5.0–8.9)	8.7 (6.2–10.3)	**0.021**
VAT (kg)	0.96 (0.53–2.29)	1.25 (0.76–1.85)	1.1 (0.89–2.60)	0.411
HOMA-IR	1.21 (0.84–1.95)	1.68 (1.49–2.04)	4.00 (2.78–5.55)	**<0.001**
				
*Adults*				
*n*	44	44	44	
FM (kg)	20.9 (14.6–27.5)	19.5 (15.5–24.6)	22.5 (15.1–30.6)	0.519
FFM (kg)	58.2 (46.4–68.4)	51.3 (43.0–59.8)	53.1 (44.2–64.4)	0.051
Adipose tissue (kg)	18.9 (15.7–29.3)	18:3 (14.6–24.6)	21.7 (16.0–31.4)	0.277
SAT total (kg)	16.9 (13.8–24.8)	17.3 (13.7–22.1)	18.6 (14.9–27.6)	0.433
SAT arms (kg)	2.1 (1.7–2.8)	2.0 (1.6–2.4)	2.2 (1.7–2.8)	0.339
SAT trunk (kg)	7.7 (5.9–11.0)	7.1 (5.2–9.9)	8.4 (5.5–13.1)	0.380
SAT legs (kg)	6.9 (5.7–10.4)	7.3 (6.3–10.1)	8.3 (6.5–10.8)	0.643
VAT (kg)	2.07 (1.32–3.59)	1.57 (0.75–2.39)	2.39 (1.23–4.90)	**0.013**
HOMA-IR	1.39 (1.06–1.66)	1.84 (1.63–2.28)	3.75 (2.67–4.88)	**<0.001**

Abbreviations: FM, fat mass; FFM, fat-free mass; SAT, subcutaneous adipose tissue; VAT, visceral adipose tissue.

(1), pre- and intrapubertal children; (2), postpubertal adolescents; (3), young adults (18–30 years old); (4), adults (30–60 years old).

Group 1: HOMA-IR/BMI ↓, residuals (1)<−0.48, (2)<−0.90, (3)<−0.92, (4)<−0.69; Group 2: HOMA-IR/BMI=, residuals (1)⩽0.16 and ⩾−0.48, (2) ⩽0.25 and ⩾−0.90, (3) ⩽0.00 and ⩾−0.92, (4) ⩽0.29 and ⩾−0.69; Group 3: HOMA-IR/BMI ↑, residuals (1) >0.16, (2) >0.25, (3) >0.00, (4) >0.29. Significant correlation coefficients in bold (Kruskal-wallis test with bonferroni correction, *P*<0.05)
